# The role of oxygen in the formation of fluorescent advanced glycation end products from α-dicarbonyl compounds

**DOI:** 10.1007/s00391-026-02662-2

**Published:** 2026-10-19

**Authors:** Parisa Ghaffari Makhmalbaf, Andreas Simm

**Affiliations:** Department of Cardiac Surgery, University Hospital Halle, University of Halle, Ernst Grube Straße 40, 06120 Halle, Germany

**Keywords:** Diabetic complications, Glyoxal, Methylglyoxal, Non-enzymatic glycation, Hypoxia, Diabetische Komplikationen, Glyoxal, Methylglyoxal, Nichtenzymatische Glykierung, Hypoxie

## Abstract

**Background:**

Advanced glycation end products (AGEs) are heterogeneous compounds that can be endogenously generated and can also be exogenously consumed through heated food. The AGEs accumulate in cells and tissues over time, increasing oxidative stress and thereby contributing to the aging process via inflammation. The physiological oxygen concentration in tissues is significantly lower than atmospheric oxygen (~21%); however, most experimental studies have been conducted under atmospheric conditions rather than physiologically relevant oxygen levels.

**Objective:**

This study aims to assess the oxygen-dependent formation of AGEs induced by glyoxal (GO) and methylglyoxal (MGO) in vitro.

**Material and methods:**

In this study, the AGE-associated fluorescence intensity was measured in human serum albumin (HSA) incubated with GO or MGO under different oxygen concentrations using a CLARIOstar plate reader. Fluorescent AGEs were quantified using excitation wavelengths of 330/360 nm and emission wavelengths of 405/440 nm. Non-fluorescent AGEs, such as carboxymethyl lysine (CML) and methylglyoxal-derived hydroimidazolone1 (MG-H1), were detected via immunoblotting.

**Results:**

The formation of AGEs increased following incubation of HSA with GO or MGO, showing clear concentration-dependent and time-dependent patterns. No statistically significant differences were observed among the tested oxygen conditions.

**Conclusion:**

These findings suggest that under the experimental conditions used in this in vitro study, glycation did not significantly differ across the tested oxygen conditions. Further investigation is warranted to explore the impact of oxygen on glycation in vivo, where additional regulatory mechanisms could play a role.

**Supplementary Information:**

The online version of this article (https://doi.org/10.1007/s00391-026-02662-2) contains supplementary material, which is available to authorized users.

## Abstract (de)

**Hintergrund:**

Fortgeschrittene Glykationsendprodukte (Advanced Glycation End Products, AGEs) sind heterogene Verbindungen, die sowohl endogen gebildet als auch exogen über erhitzte Lebensmittel aufgenommen werden können. Sie reichern sich im Laufe der Zeit in Zellen und Geweben an, erhöhen den oxidativen Stress und tragen über entzündliche Prozesse zum Alterungsprozess bei. Die physiologische Sauerstoffkonzentration im Gewebe liegt deutlich unter der atmosphärischen Sauerstoffkonzentration (~21 %). Dennoch wurden die meisten experimentellen Studien unter atmosphärischen Bedingungen und nicht unter physiologisch relevanten Sauerstoffkonzentrationen durchgeführt.

**Zielsetzung:**

Ziel dieser Studie war es, die sauerstoffabhängige Bildung von AGEs, induziert durch Glyoxal (GO) und Methylglyoxal (MGO), in vitro zu untersuchen.

**Material und Methoden:**

In dieser Studie wurde die AGE-assoziierte Fluoreszenzintensität in Humanserumalbumin (HSA) gemessen, das unter verschiedenen Sauerstoffkonzentrationen mit GO oder MGO inkubiert wurde, unter Verwendung eines CLARIOstar-Plattenlesegeräts. Fluoreszierende AGEs wurden bei Anregungswellenlängen von 330/360 nm und Emissionswellenlängen von 405/440 nm quantifiziert. Nichtfluoreszierende AGEs wie CML und MG-H1 wurden mittels Immunoblotting nachgewiesen.

**Ergebnisse:**

Die AGE-Bildung nahm nach der Inkubation von HSA mit GO oder MGO zu und zeigte dabei deutliche konzentrations- und zeitabhängige Muster. Zwischen den untersuchten Sauerstoffbedingungen wurden keine statistisch signifikanten Unterschiede festgestellt.

**Schlussfolgerung:**

Diese Ergebnisse deuten darauf hin, dass sich die Glykation unter den in dieser In-vitro-Studie verwendeten Versuchsbedingungen bei den untersuchten Sauerstoffbedingungen nicht signifikant unterschied. Es sind weitere Untersuchungen erforderlich, um den Einfluss von Sauerstoff auf die Glykation in vivo zu untersuchen, wo möglicherweise zusätzliche Regulationsmechanismen eine Rolle spielen.

## Introduction

**Fig. 1:**
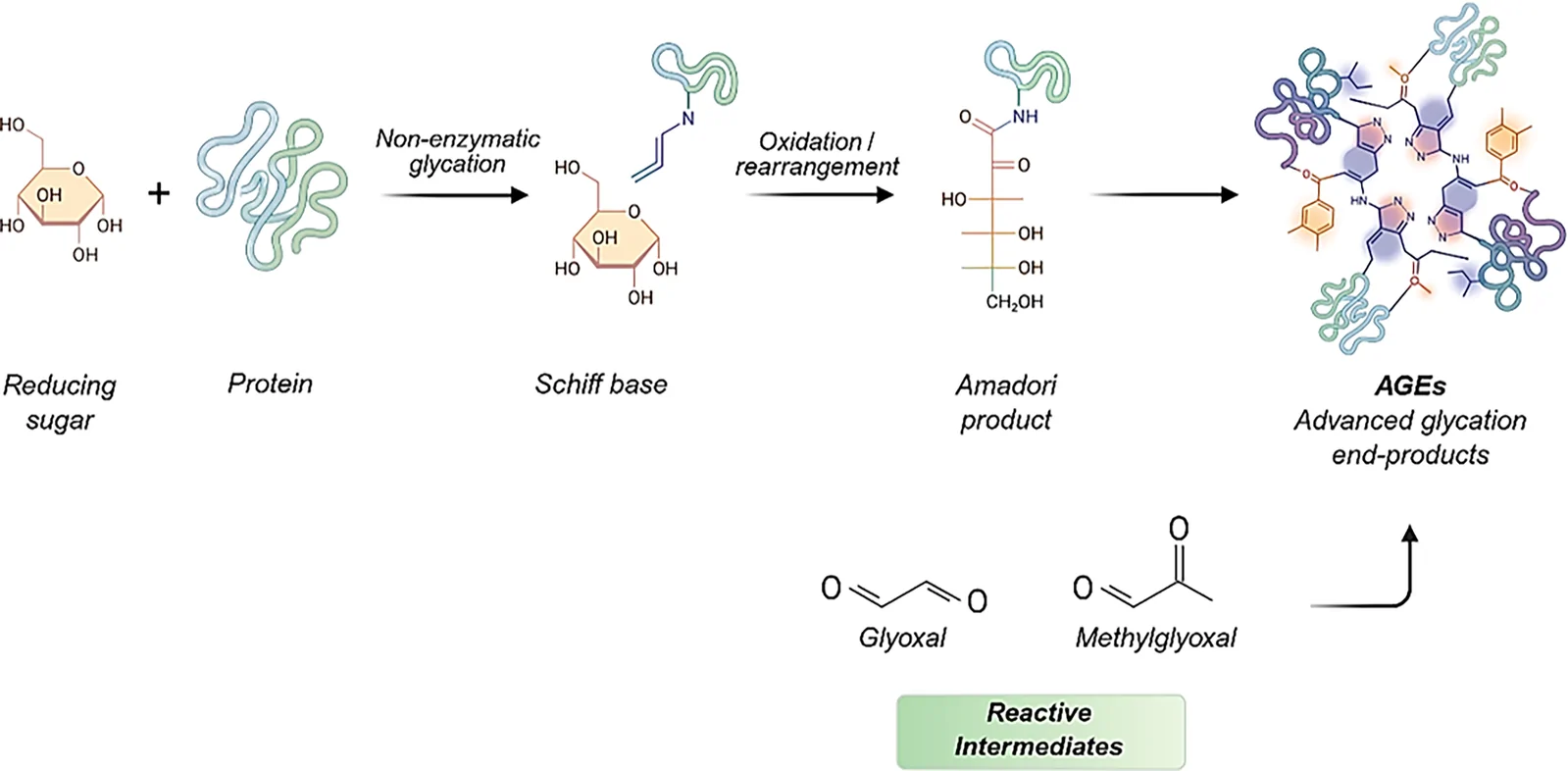
Formation of advanced glycation end-products (AGEs). The schematic illustrates the formation of AGEs from reducing sugars via nonenzymatic glycation, progressing through Schiff base and Amadori intermediates. Reactive dicarbonyls such as glyoxal and methylglyoxal contribute to carbonyl stress and promote AGE formation. Created with BioRender.com

The Maillard reaction, first described by Louis Camille Maillard in 1912, is a nonenzymatic reaction between amino acids and reducing sugars that produces brown pigments [1]. This process, also termed nonenzymatic glycation or browning reaction primarily targets lysine and arginine residues in proteins. As the product of this reaction, the advanced glycation end products (AGE), is an irreversible modification, its elimination relies solely on protein degradation and subsequent replacement. The initial stage of the nonenzymatic reaction generates a labile intermediate, which then undergoes nonenzymatic stabilization to form an irreversible protein modification [2]. Following glycation, the protein has posttranslational modifications classified as AGEs. Figure 1 illustrates the formation of AGEs. These products and their chief cellular receptor, RAGE, participate in multiple signalling pathways, including nuclear factor kappa B (NF-κB), c-Jun N-terminal kinase (JNK), transforming growth factor beta (TGF‑β), mitogen-activated protein kinases (MAPKs) and extracellular signal-regulated kinases 1/2 (ERK1/2). As a result, they contribute to enhanced oxidative stress and inflammation, which play pivotal roles in cardiovascular and neurodegenerative diseases, diabetes and other age-related conditions [3–5]. Glyoxal (GO) and methylglyoxal (MGO) are reactive dicarbonyl metabolites derived from metabolic pathways and serve as major precursors in the formation of AGEs. Whereas oxidative stress is known to induce protein glycation, little is known about the role of oxygen concentration.

Oxygen regulation in cardiac tissue is important to support myocardial function and cellular balance. Although atmospheric oxygen is ~21%, physiological oxygen levels in mammalian tissues are much lower and vary considerably across organs, vascular compartments, and local cellular microenvironments. Consequently, conventional cell-culture conditions may not accurately reproduce the oxygen milieu to which cells are exposed in vivo [6, 7]. Disruption of redox homeostasis and pathological remodelling, which can result from oxidative stress or hypoxia, can occur with alterations in oxygen levels. Hypoxia, in particular, triggers wide-ranging molecular and cellular adaptations, including changes in metabolism, erythropoiesis, angiogenesis, neurogenesis, mitochondrial trafficking and autophagy [8].

In cardiovascular disease (CVD), ischemia (low oxygen) may modify AGE formation or reactive carbonyl production. This interplay between glycation and oxygen imbalance may contribute to inflammation, metabolic disruption and pathological remodelling in the heart. Several studies have shown that AGEs contribute to cardiovascular dysfunction [9, 10]; however, it is still unclear exactly how AGEs affect the cardiovascular system.

The aim of this study was to examine whether the glycation reaction is modulated by oxygen, emphasizing the roles of glyoxal (GO) and methylglyoxal (MGO) in the formation of AGEs.

## Material and methods

### Chemicals and reagents

Glyoxal and methylglyoxal were purchased from Sigma-Aldrich (Taufkirchen, Germany) as a 40% aqueous solution. The 0.01 M working solution was prepared by diluting 8.8 M and 6.5 M MGO/GO with ultrapure water. Human serum albumin (HSA) fraction V, was obtained from Bio&Sell (Feucht, Germany) and dissolved in phosphate-buffered saline (PBS) to prepare a 10 mg/ml stock solution. The PBS tablets from Gibco, Thermo Fisher Scientific (UK), were used to prepare 1× PBS (PH 7.4).

### Preparation of reaction media

The human serum albumin (10 mg/ml) in PBS was glycated through incubation with glyoxal or methylglyoxal at 37 C° in a humidified atmosphere. To assess the effect of oxygen on the reaction, incubations were performed at nominal gas-phase O_2_ setpoints of 1%, 5%, or 21%. As dissolved oxygen concentrations in the reaction mixtures were not directly measured, these values refer to the controlled gas phase O_2_ conditions in the reader chamber and do not represent verified dissolved O_2_ concentrations in the wells.

### Fluorescence spectroscopy

The kinetics of AGE formation were assessed via fluorescence spectroscopy using a CLARIOstar® Plus microplate reader (BMG Labtech, Ortenberg, Germany) equipped with an atmospheric control unit for independent regulation of O_2_ and CO_2_ within the reader chamber. Prior to inserting the microplate, the instrument atmosphere was equilibrated to the desired oxygen level (1%, 5%, or 21%) by replacing ambient air with nitrogen while maintaining 5% CO_2_ and 37 °C. Reaction mixtures were prepared under normoxic conditions and immediately transferred to the pre-equilibrated reader. During the 48h kinetic measurement, the microplate remained inside the reader and O_2_, CO_2_ and temperature were continuously controlled by the CLARIOstar Plus, with fluorescence readout acquired every 2 h over a 48- h incubation period. The measurements were performed on a 96-well plate, black walled, clear bottom (Greiner Bio-One, Germany), with a fixed HSA concentration of 5 mg/ml. Formation of AGEs was detected by measuring their intrinsic fluorescence at two standard wavelength pairs: an excitation/emission of 330/405 nm and 360/440 nm. Data were processed in Microsoft Excel, and statistical analysis and graphing were performed using GraphPad Prism (11.0.1; GraphPad Software, Boston, MA, USA).

### Dot blot analysis

In this study, nonfluorescent AGEs, specifically carboxymethyl lysine (CML) and methylglyoxal-derived hydroimidazolone1 (MG-H1), were detected using dot blot immunoassay. Human serum albumin (HSA) was incubated with glyoxal (GO) or methylglyoxal (MGO) for 48 h. Following incubation, samples (5 mg/ml) were diluted 1:1 with PBS to achieve a final concentration of 2.5 mg/ml. A volume of 100 µl from each sample was then applied directly onto a 0.4 µm nitrocellulose membrane using a Whatman Minifold I Slot Blot System, followed by washing with PBS according to the manufacturer’s instructions. After air drying, total protein was assessed using the REVERT 700 Total Protein Stain Kit (LI-COR) to ensure equal loading. Membranes were subsequently blocked with 5% skimmed milk in TBST and incubated with primary antibodies against CML26 (mouse monoclonal, Abcam ab125145) and MG-H1 (mouse monoclonal, Cell Biolabs, STA-011). Following primary antibody incubation, membranes were incubated with an IRDye-conjugated anti-mouse secondary antibody. Fluorescent signals were detected using the LI-COR Odyssey imaging system, and images were acquired in the 700 nm and 800 nm channels. Dot blot experiments were performed in three independent experiments. Within each independent experiment, 2–3 technical replicate spots per condition were included on the same membrane. Technical replicate intensities were averaged, and the resulting mean value was treated as one independent experiment for statistical analysis. Signal intensities were quantified using Image Studio software with identical analysis settings applied to all membranes and were normalized to total protein staining to account for differences in protein loading. Membranes were processed using the same antibody incubation, washing, and imaging conditions. Quantification was not performed in a blinded manner.

### Statistical analysis

The measurements were performed in at least three technical replicates for each sample. The statistical data were processed based on 5 independent experiments for fluorescence measurements and 3 independent experiments for dot blot analyses. The data are presented as a mean ± standard deviation (SD). Statistical significance between experimental groups was assessed using one-way ANOVA, two-way ANOVA or two-way repeated measure ANOVA followed by Tukey’s multiple comparison test (GraphPad Prism, 11.0.1). A *p*-value less than 0.05 was considered statistically significant.

## Results

### Concentration dependency of AGEs formation

**Fig. 2:**
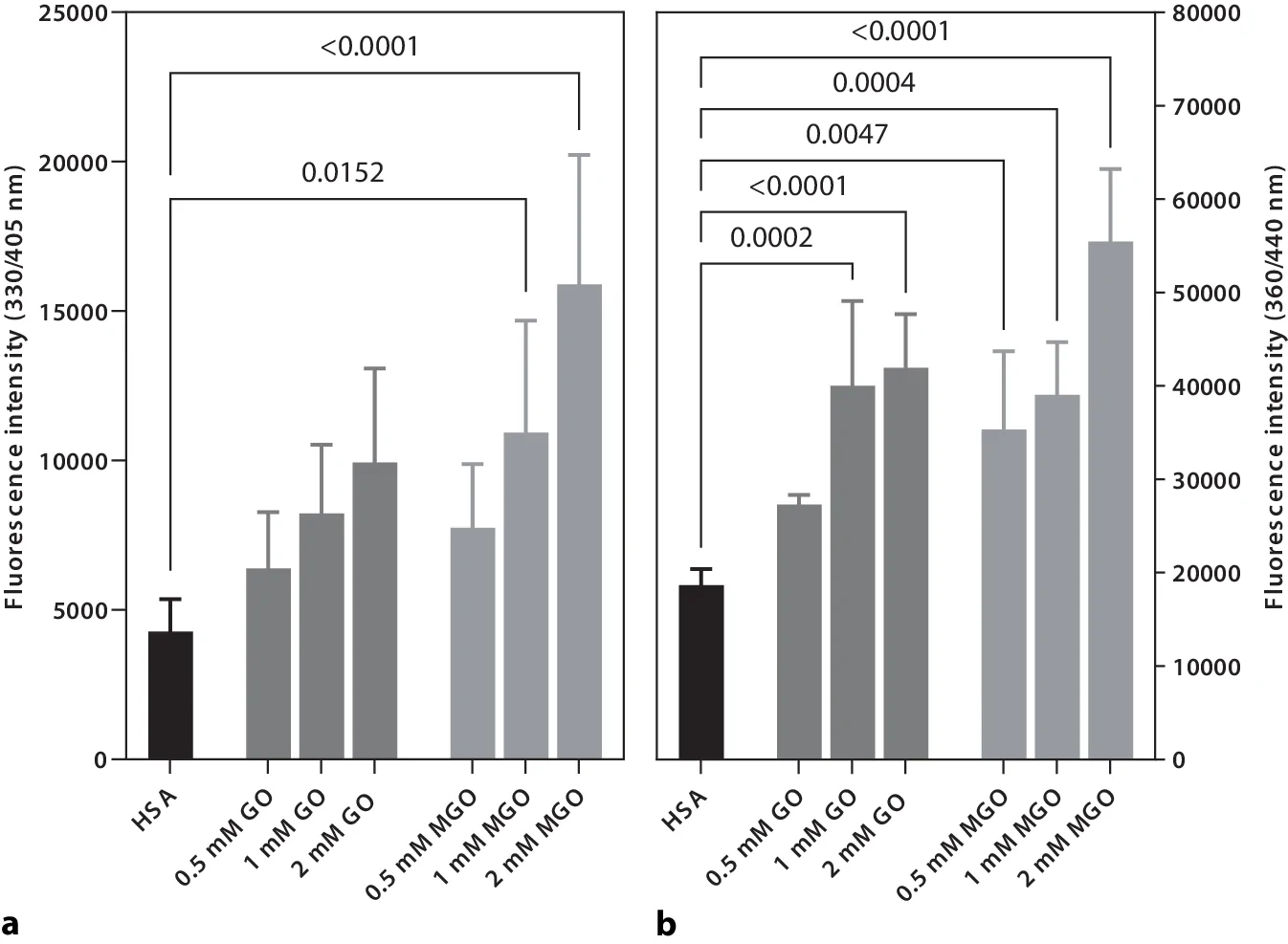
Advanced glycation end-products (AGEs) formation in human serum albumin (HSA). HSA was incubated for 48 h at 21% O_2_ with glyoxal (GO; 0.5–2 mM) or methylglyoxal (MGO; 0.5–2 mM). Fluorescence intensity was recorded at 330/405 nm (**a**) and 360/440 nm (**b**). Bars represent mean ± SD; data were analyzed by one-way ordinary ANOVA (*n* = 5 independent experiments). Exact *P* values are shown above the brackets

First, we analyzed the formation of AGEs and HSA was incubated for 48 h at 1%, 5%, and 21% O_2_ with glyoxal (GO) or methylglyoxal (MGO) at concentrations ranging from 0.5–2 mM. Formation of AGEs was assessed by measuring fluorescence intensities at 330/405 nm and 360/440 nm. In the 21% O_2_ condition, both GO and MGO induced a dose-dependent increase in fluorescence, with MGO showing higher intensities overall (Fig. 2a, b). We observed similar dose-dependent trends under 5% and 1% O_2_.

### Time dependency of AGEs formation

**Fig. 3:**
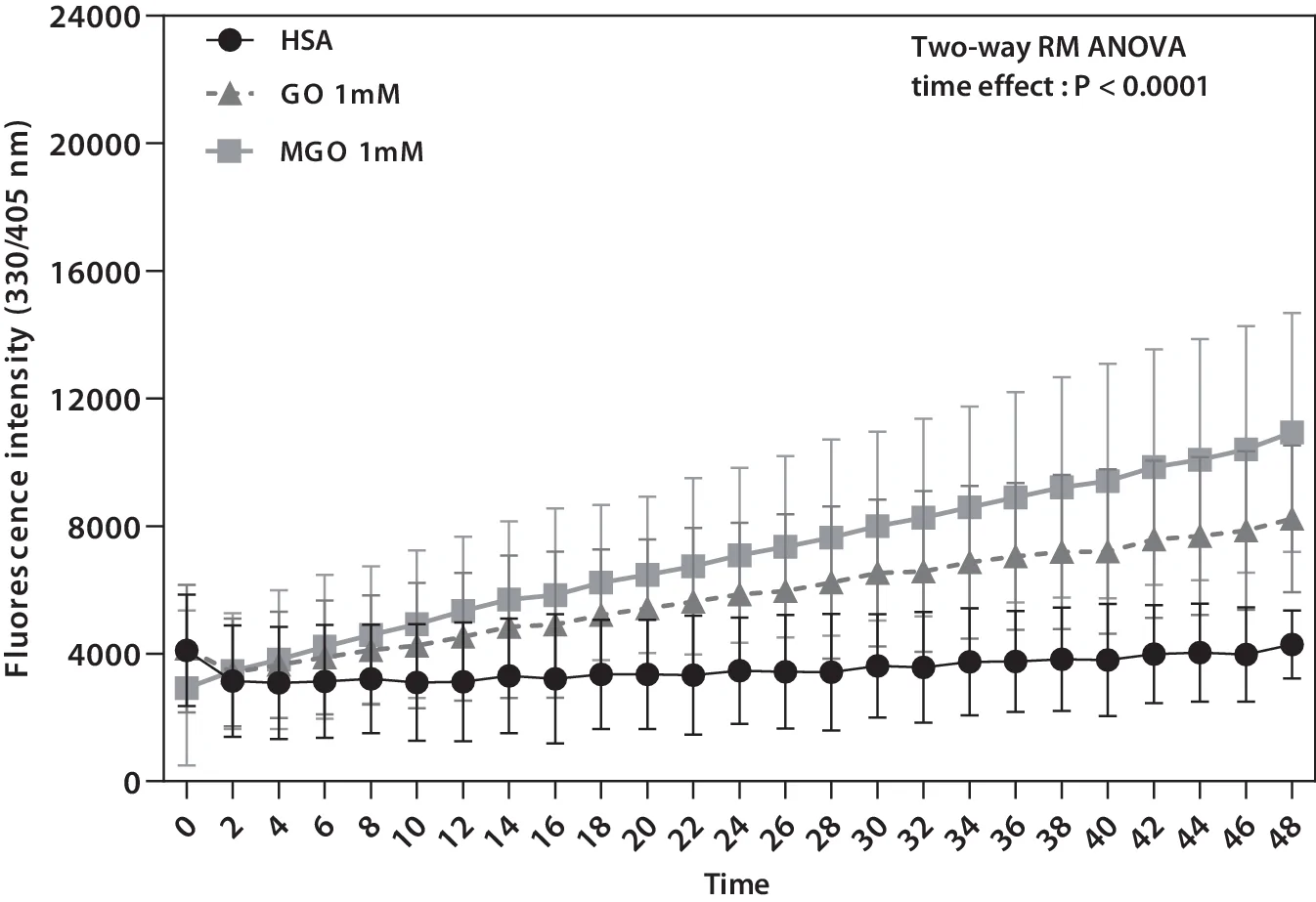
Time-dependent formation of advanced glycation end-products (AGEs) in human serum albumin (HSA) incubated at 21% O_2_ with 1 mM glyoxal (GO) or 1 mM methylglyoxal (MGO) for up to 48 h. Fluorescence intensity at 330/405 nm was recorded at the indicated time points. Data are shown as mean ± SD; *n* = 5 independent experiments. Two-way repeated-measures ANOVA revealed a significant effect of time (*p* < 0.0001)

To determine the time-dependent formation of AGEs, HSA was incubated with 1 mM GO or 1 mM MGO for up to 48 h at 1%, 5%, and 21% O_2_. Formation of AGEs was monitored by fluorescence intensity at 330/405 nm. In 21% of O_2_, both GO and MGO induced a progressive increase in fluorescence over time, with MGO showing a more pronounced effect compared to GO and native HSA. Two-way repeated measures ANOVA, time effect (*P* < 0.0001), together with a significant time × treatment interaction (*P* = 0.0001), indicating that GO and MGO induced distinct time-dependent increases in AGEs fluorescence compared with HSA alone, whereas the treatment main effect was not significant (*P* = 0.0818). Figure 3 displays only 1 mM GO/MGO at 21% O_2_. Detailed statistical results are provided in Supplementary Data 1.

### Oxygen dependency of AGEs formation

**Fig. 4:**
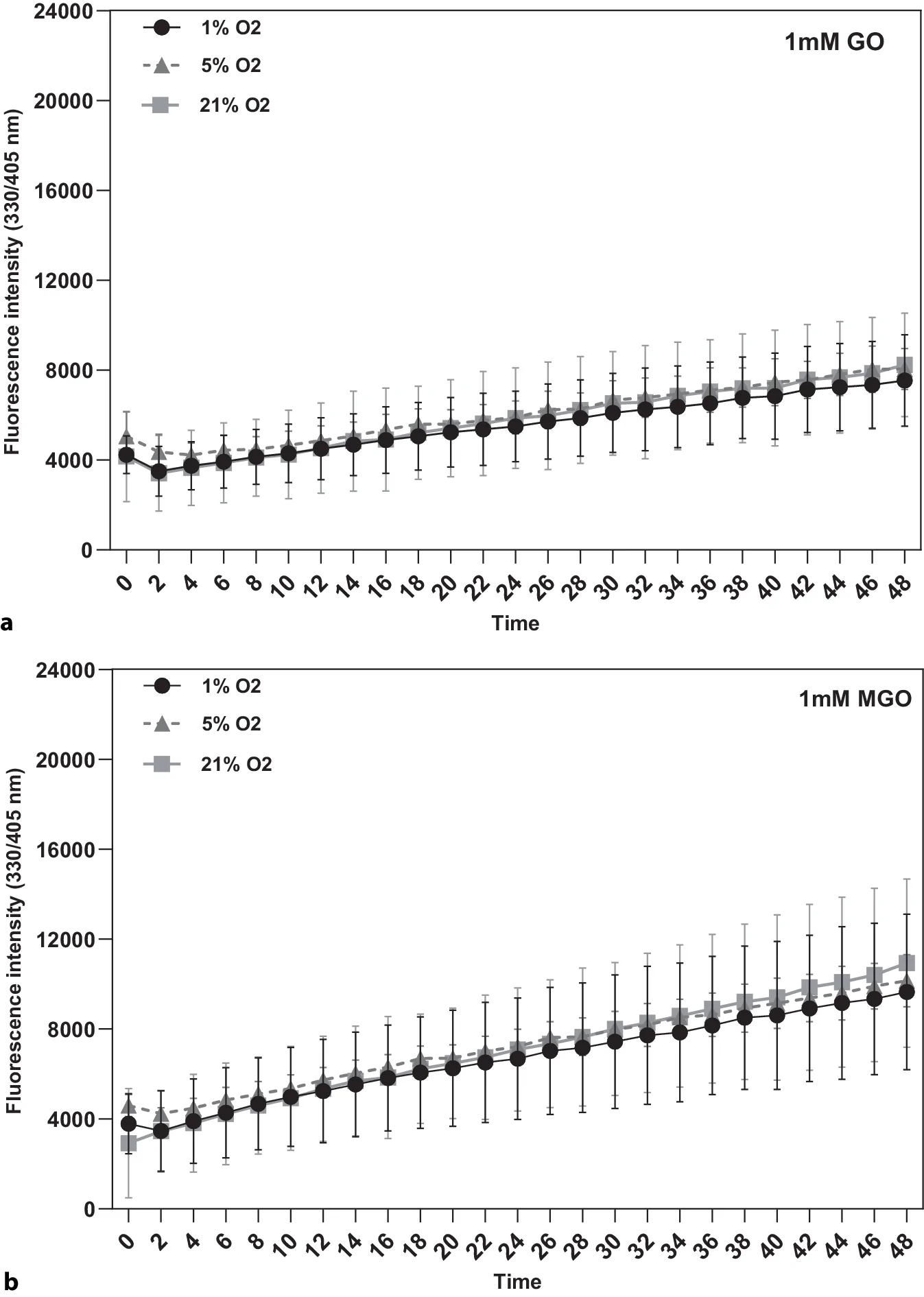
Fluorescence intensity of AGE-modified human serum albumin incubated with 1 mM glyoxal or methylglyoxal under 1%, 5% or 21% O_2_ for 48 h. Fluorescence was recorded at 330/405 nm repeatedly over time, and curves represent mean ± SD (*n* = 5) for each oxygen condition. Two-way repeated-measures ANOVA revealed a robust increase in fluorescence over time, whereas oxygen concentration did not significantly affect the time course or endpoint AGE-associated fluorescence. **a** GO, **b** MGO

To assess whether glyoxal (GO)-induced or methylglyoxal (MGO)-induced glycation is oxygen dependent, fluorescence intensity was measured in HSA incubated with 1 mM GO or 1 mM MGO over 48 h under 3 oxygen conditions: 1%, 5%, and 21% O_2_. For 1 mM GO, two-way repeated measures ANOVA revealed a robust increase in fluorescence over time, main effect of time: (F (1.738, 20.85) = 186.7, *P* < 0.0001), indicating progressive AGE accumulation but no statistically significant main effect of oxygen (F (2, 12) = 0.1249, *P* = 0.8837) and no statistically significant time × oxygen interaction (F (3.476, 20.85) = 0.8800, *P* = 0.4806). To further quantify the oxygen effect at the 48 h endpoint, we examined pairwise comparisons between oxygen tensions, reporting mean differences with their 95% confidence intervals and adjusted *P*-values. All three comparisons (1% vs. 5%, 1% vs. 21% and 5% vs. 21% O_2_) showed small mean differences, wide 95% confidence intervals spanning zero and high adjusted *P*-values (~ 0.8–0.9). For 1 mM MGO, two-way repeated-measures ANOVA likewise showed a significant main effect of time (F(1.317, 15.81) = 117.9, *P* < 0.0001) but no statistically significant oxygen main effect of oxygen (F (2,12) = 0.07351, *P* = 0.9295) and no statistically significant time × oxygen interaction (F (2.634, 15.81) = 0.8278, *P* = 0.4845) (figure 4a, b). Detailed statistical results for all time points are provided in Supplementary Data 1.

### CML and MG-H1 formation induced by GO or MGO and its modulation by oxygen levels

**Fig. 5:**
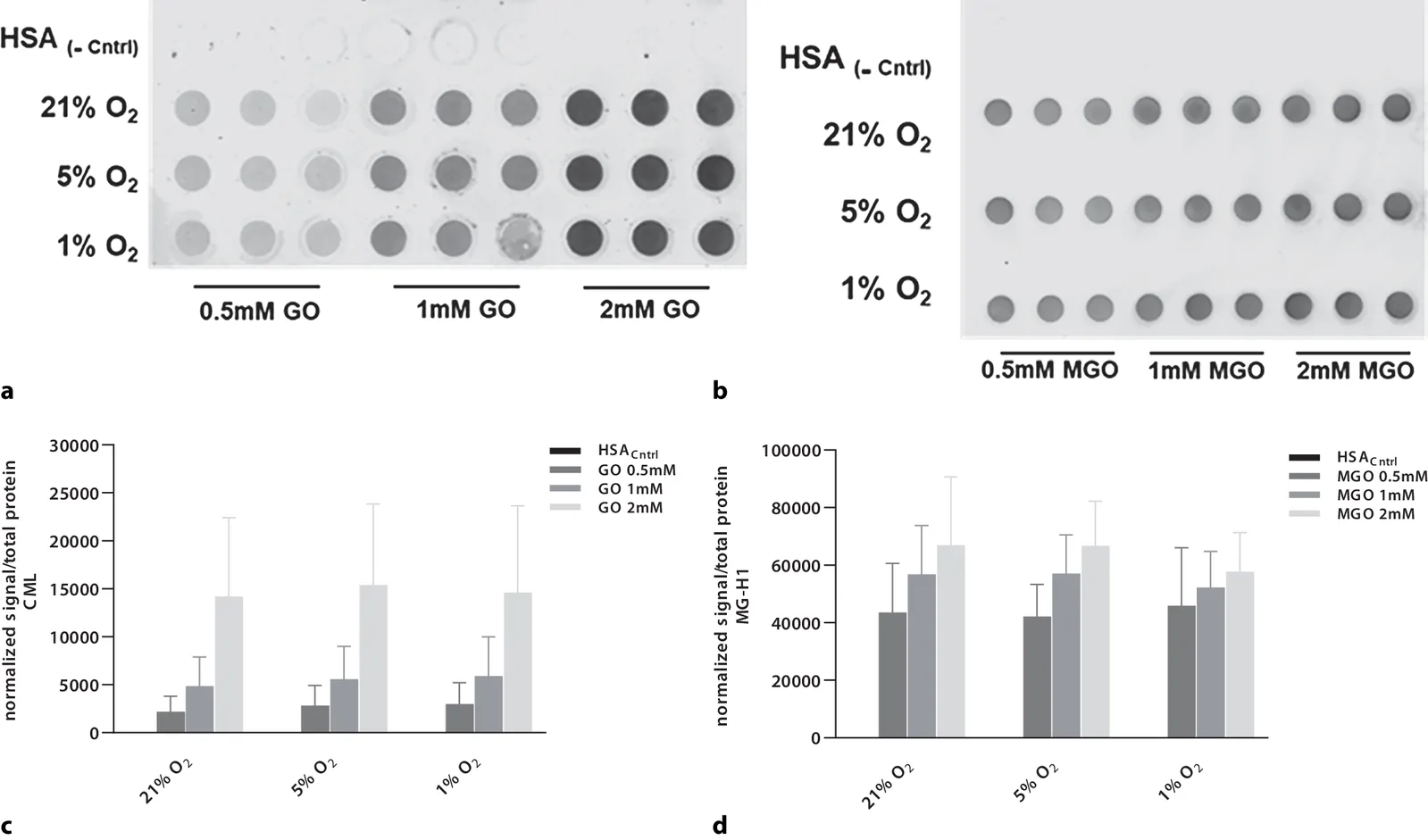
Quantification of CML (**a**) and MG-H1 (**b**) dot blots, and corresponding densitometric analyses for CML (**c**) and MG-H1 (**d**), normalized to total protein under different oxygen concentrations (21%, 5%, and 1% O_2_) following HSA control, GO or MGO treatment (0.5, 1, and 2 mM). Data are presented as mean ± SD. Two-way ANOVA followed by multiple comparisons revealed no statistically significant differences between groups (ns, *p* > 0.05)

HSA was incubated with glyoxal (GO) or methylglyoxal (MGO) at concentrations of 0.5, 1, and 2 mM under 3 oxygen conditions (21%, 5%, and 1% O_2_) for 48 h. Dot blot assays were performed to assess nonfluorescent protein modifications induced by GO (CML) and MGO (MG-H1). Signal intensity increased with higher GO and MGO concentrations. Carboxymethyl lysine (CML) and methylglyoxal-derived hydroimidazolone1 (MG-H1) levels were assessed by dot blot and normalized to total protein. Despite the concentration-dependent increase in GO and MGO-induced signal intensity, no statistically significant differences were observed across oxygen conditions (Fig. 5a–d). Detailed statistical results are provided in Supplementary Data 1.

## Discussion

Advanced glycation end products (AGEs) can modify a wide range of extracellular and intracellular proteins, thereby altering their structure and function. Known AGE targets include serum albumin, hemoglobin, type IV collagen, histones, and key metabolic and regulatory proteins [11–14]. In addition to structural modification, AGEs can promote intracellular oxidative stress and inflammation via NADPH oxidase and NF-κB. Several signalling pathways, such as phosphoinositide 3-kinase/protein kinase B (PI3K/Akt) and Janus kinase/signal transducer and activator of transcription (JAK/STAT) pathway, and mitogen-activated protein kinases (MAPKs), are associated with the simple ligand receptor interaction which have been implicated in propagating its downstream effect [15, 16]. Advanced glycation end products (AGEs) can form covalent cross-links with proteins, leading to the structural modifications that may disrupt normal protein function. These cross-linked complexes may reduce proteins turnover and contribute to oxidative stress. Consequently, AGE accumulation has been implicated in metabolic dysregulation and in the development of diabetic complications [17]. From a vascular perspective, such chemical modifications are expected to accumulate over time and reshape the protein network of endothelial cells.

Hypoxia, a physiological condition which is characterized by reduced oxygen availability, can trigger a variety of molecular and cellular responses, including changes in energy metabolism, erythropoiesis, angiogenesis, neurogenesis, mitochondrial dynamics, and autophagy [8]. Endothelial cells are especially sensitive to such fluctuations in oxygen tension. In addition, hypoxic tumor cells undergo a profound metabolic reprogramming from mitochondrial respiration to anaerobic glycolysis, and this glycolytic bias increases the production of reactive dicarbonyls. As a result, AGEs accumulate within hypoxic tumor regions, a phenomenon with important implications for tumor growth and therapeutic resistance [18, 19]. In models of myocardial ischemia-reperfusion (I/R), activation of the AGE–RAGE axis is increased in ischemic cardiac tissue. This activation alters myocardial metabolism and function during ischemia-reperfusion. Consistent with this concept, RAGE inhibition in such models has been shown to attenuate tissue damage and improve myocardial function and energy metabolism [20]. Previous studies have shown higher AGE accumulation in endothelial cells under hypoxic conditions, suggesting a potential link between oxygen availability and glycation [21]; however, it remains unclear to what extent this association reflects a direct oxygen dependency of the glycation chemistry itself or a consequence of hypoxia-driven cellular and metabolic responses, such as enhanced production of reactive carbonyl species and oxidative stress. This study investigated whether the glycation reaction, driven by reactive dicarbonyls and independent of cellular context, is naturally oxygen dependent. We assessed the in vitro mechanism of glycation by analyzing the formation of AGEs in the presence of different concentrations of GO and MGO under different oxygen levels. In these controlled conditions, oxygen availability did not significantly influence AGE formation; however, because dissolved oxygen concentrations in the reaction mixture were not directly measured, these findings should be interpreted in the context of the nominal gas phase O_2_ conditions controlled in the reader chamber, rather than as effects of directly verified dissolved oxygen concentrations.

## Conclusion

Our findings suggest that under the experimental conditions used in this study, glycation did not differ significantly across the tested oxygen concentrations in vitro; however, these findings do not exclude a possible effect of oxygen on glycation and it is important to remember that these systems are simplified and controlled and do not reflect the full complexity of living organisms. For this reason, the results should be interpreted with caution and taking the limits of the model into account. In vivo, many physiological, metabolic, and immune processes interact and can strongly affect AGE accumulation and hypoxia-associated glycation effects. In cells and living organisms, AGE accumulation may be affected by upstream biological processes influenced by oxygen availability. More research using realistic in vivo models is needed to better understand how oxygen influences AGE-related disease via glycation. In particular, models that enable the interaction between oxygen levels, dicarbonyl stress, and endothelial responses should be evaluated over the long term.

## Supplementary Information

ESM1: Supplementary material 1

## Data Availability

All data supporting the findings of this work are available from the corresponding author upon reasonable request.

## Abbreviations

AGEs: Advanced glycation end products
CML: Carboxymethyl lysine
CVD: Cardiovascular disease
ERK1/2: Extracellular signal-regulated kinases 1/2
GO: Glyoxal
HSA: Human serum albumin
JAK/STAT: Janus kinase/signal transducer and activator of transcription
JNK: c-Jun N-terminal kinase
MAPKs: Mitogen-activated protein kinases
MG-H1: Methylglyoxal-derived hydroimidazolone1
MGO: Methylglyoxal
NF-κB: Nuclear factor kappa B
PBS: Phosphate-buffered saline
PI3K/Akt: Phosphoinositide 3-kinase/protein kinase B
RAGE: Receptor for advanced glycation end products
ROS: Reactive oxygen species
TBST: Tris-buffered saline with Tween-20
TGF-β: Transforming growth factor beta

## Declarations

The authors used Grammarly and ChatGPT solely for language editing and improving readability. The authors reviewed and verified all scientific content, data, analyses, results, and conclusions and take full responsibility for the final manuscript.
